# Method development for pancreatic and ovarian cancer baseline ctDNA detection and measurable residual disease monitoring

**DOI:** 10.12688/openreseurope.23403.1

**Published:** 2026-04-03

**Authors:** Tim Alexander Steiert, Philipp M. Altrock, Axel Künstner, Vishal Dixit, Burkhard Brandt, Pamela Pinzani, Hauke Busch, Andre Franke, Michael Forster

**Affiliations:** 1 Institute of Clinical Molecular Biology, Christian-Albrechts-University of Kiel & University Hospital Schleswig-Holstein, Kiel, Schleswig-Holstein, 24118, Germany; 2 Department of Hematology and Oncology, Christian-Albrechts-University of Kiel & University Hospital Schleswig-Holstein, Kiel, Schleswig-Holstein, 24105, Germany; 3 Medical Systems Biology Group, Lübeck Institute of Experimental Dermatology, University of Lübeck, Lübeck, Schleswig-Holstein, 23538, Germany; 4 Twist Bioscience, Inc., South San Francisco, California, 94080, USA; 5 Institute of Clinical Chemistry, University Medical Center Schleswig-Holstein, Kiel, Schleswig-Holstein, 24105, Germany; 6 Department of Experimental and Clinical Biomedical Sciences, University of Florence and Molecular and Clinical Biochemistry Unit, Careggi University Hospital, Florence, Tuscany, 50139, Italy

**Keywords:** Liquid biopsy, Measurable residual disease, Minimal residual disease, Personalized medicine, Precision medicine, Low-level cancer variant allele frequency detection

## Abstract

**Background:**

Pancreatic cancer and ovarian cancer are very challenging to diagnose at early stages. The endoscopic retrieval of biopsy tissue from a suspected benign or malignant lesion is challenging due to the tissue’s nature. Therefore, within the Instand-NGS4P framework, we developed Measurable Residual Disease (MRD) prototypes to analyze blood plasma samples, with the aim of cost-effectively supporting the differential diagnosis of suspected pancreatic or ovarian neoplasms.

**Methods:**

Our MRD prototypes examine blood plasma for mutations in cell-free DNA in specific genes associated with pancreatic neoplasms or ovarian neoplasms, respectively. Unique molecular identifiers (UMIs) are used to enable bioinformatic error correction. Ultra-deep sequencing is demonstrated on sequencing platforms from two different vendors (Illumina and MGI). We provide detailed information on bioinformatic processing of sequencing data to perform error-correction.

**Results:**

Using commercially available reference standards, we demonstrate stable mutation detection down to a variant allele frequency (VAF) of 0.1%. At a coverage of 4,000x duplex consensus reads, only two false positives were observed, which can be efficiently mitigated using an appropriate filtering strategy.

**Conclusions:**

The technical usability of our MRD prototype has been clearly demonstrated for stable low-level VAF detection in commercial reference samples.

## Plain language summary

Early detection of pancreatic and ovarian cancer is difficult, and tissue biopsies are often hard to obtain. We developed blood-based test prototypes that detect tiny amounts of cancer DNA using highly sensitive sequencing. Using reference samples, we reliably detected mutations at very low levels (0.1%) with few false signals. These results show that the approach is technically robust and suitable for low-level blood-based cancer testing.

## 1. Introduction

Measurable or Minimal Residual Disease (MRD) is a term originating from leukemia diagnostics of blood or bone marrow samples. In the 1980s, when the term MRD was coined, the technologies used for detection and disease surveillance were rather insensitive. Accurate and sensitive (≤0.01% or ≤10 ^−4^) detection was first possible using polymerase chain reaction (PCR) approaches that targeted specific genetic aberrations or translocations in Acute Lymphoblastic Leukemia (ALL), ^1^ describing “molecular remission” that proved to be a superior indicator than clinical parameters or cell morphology. Today, modern next-generation sequencing (NGS) or next-generation flow techniques have further improved MRD detection, eliminating the need for *a priori* definition of specific molecular markers.

Following MRD in leukemia, the NGS analysis of circulating tumor DNA (ctDNA) led to the broad extension of MRD to solid tumors. ^2^ Subsequent trials generalized this framework to several solid tumor entities, *e.g.* non-small cell lung cancer, ^3^ breast cancer, ^4^ melanoma, ^5^ urinary-tract cancers, ^6^ prostate, ^7^ and pancreatic cancers. ^8^ Just these mentioned entities account for more than 40% of all cancer-related deaths within the EU-27. ^9^ In all these entities, ctDNA positivity after surgery or systemic therapy usually constitutes an early molecular herald of relapse. Despite this, the number of CE-/IVDR-certified, MRD-approved ctDNA assays in the European Union remains very low.

In 2019, the EU Horizon 2020 project called for the first-ever pre-commercial procurement (PCP) proposals addressing the topic *SC1-BHC-10-2019 - Innovation Procurement: Next Generation Sequencing (NGS) for routine diagnosis.* Two proposals were awarded, one of which was Instand-NGS4P (Grant agreement ID: 874719).

The two patient groups targeted within the PCP project Instand-NGS4P were leukemia patients and solid tumor patients. For these patient groups, the project aimed to foster the development of automatable prototype solutions for solid tumor tissue, MRD, and germline pharmacogenomic in vitro diagnostics that would be compliant with relevant regulations and technical standards. This development would be carried out by competing commercial subcontractors in accordance with the rules for EU Horizon PCP projects.

To encourage automatable innovative developments within the diagnostic workflow, the unmet needs were identified by the consortium and refined based on the results of an open market consultation; moreover, innovation challenges were defined and subdivided into four areas of the diagnostic workflow: i) pre-analytics and wet laboratory, ii) sequencing, iii) bioinformatics, and iv) clinical data interpretation, e-medication, and reporting.

To connect these four areas, our project initiated the first-ever European standardization documentation for NGS-based IVD, CEN/TS 17981–1:2023, in vitro diagnostic Next Generation Sequencing (NGS) workflows - Part 1: Human DNA examination, and CEN/TS 17981–2:2023, in vitro diagnostic Next Generation Sequencing (NGS) workflows - Part 2: Human RNA examination. At the time of writing this manuscript, these CEN/TS documents are the basis on which the international standardization documents ISO/AWI 25379–1 and ISO/AWI 25379–2 are being drafted.

In the final phase of the Instand-NGS4P project, prototype testing began in Spring 2024 and continued until the end of the project in Fall 2025. Here, we present the technical results from two MRD wet laboratory prototypes for pancreatic and ovarian cancer, applied to synthetic reference samples with known low-level mutation levels (variant allele frequency, VAF). The prototypes were developed within Instand-NGS4P by the subcontractor consortium TWIST/Platomics/MGI (TPM). Instand-NGS4P specifications included the use minimal volumes of blood sample, the use of patient identity fingerprinting for quality control purposes, ^10, 11^ unique molecular identifiers (UMI) for bioinformatic error correction, and the improvement of mutation detection from 0.5% VAF limit of detection (LoD) to 0.1% VAF LoD or better.

From a technical perspective, for cell-free DNA (cfDNA)-based MRD assays to be clinically meaningful, they must achieve exceptionally high sensitivity and specificity. These assays are tasked with detecting extremely low levels of tumor-derived DNA among a vast background of non-tumor circulating cfDNA. A false-negative MRD result can have significant clinical implications by underestimating the disease, potentially leading to insufficient treatment and rapid disease progression. On the other hand, false-positive results, often arising from misinterpreting sequencing errors as true tumor signals, may lead to unnecessary therapeutic escalation and toxicity. Therefore, the development of robust, cost-effective assays and gene panels, as well as advanced error suppression strategies, is critical. Balancing these diametrically opposed demands will be essential for cfDNA-based MRD testing to become a standard of care in oncology.

## 2. Materials and methods

### Instand-NGS4P master panel

TPM designed and tested a master capture panel of capture probes within the Instand-NGS4P consortium, comprising 1,648 cancer genes and intronic regions for fusion gene detection with a total target region size of 7.6 Mbp. From this stock of pre-designed capture probes, customized sub-panels can be produced and delivered within a few business days.

### Instand-NGS4P pancreatic sub-panel and ovarian sub-panel

The consortium experts specified gene lists for MRD detection in pancreatic cancer ( **Table S1**) and ovarian cancer ( **Table S2**), based on publicly available data from The Cancer Genome Atlas on the most recurrently mutated gene in these cancer entities.

As an important note, extremely deep sequencing is necessary to achieve 0.1% VAF LoD. This is expensive if the panel’s target region is large. To focus on a small target region, TPM further analyzed publicly available data for cancer mutation hotspots. While performing this analysis, TPM found that publicly available data were duplicated, either because they were reported in different studies with names that did not clearly match, or because different targeted sequencing panels or different samples from the same patient were sequenced. No clear data deconvolution was feasible. To avoid the risk of skewed frequencies at mutation hotspots, it was decided to use the expert-specified gene lists and rely on frequency data from The Cancer Genome Atlas. Thus, capture probes for the entire coding regions of the genes were used in the custom panels for pancreatic cancer and ovarian cancer.

### New cfDNA pan-cancer reference standards

Newly developed Twist cfDNA Pan-Cancer Reference Standards v2 with 0%, 0.1%, and 0.5% VAF were used to iteratively determine the raw sequencing output required to detect variants in the standard with 0.1% VAF after bioinformatic error correction.

### Step 1: First explorative experiment at user site

In a first exploratory experiment at the user site UKSH, libraries were prepared as technical duplicates using 20 ng of each 0%, 0.1%, and 0.5% sample of the Twist cfDNA Pan-Cancer Reference Standards v2. The standards were frozen in tubes with 300 ng cfDNA in 15 μL. The volume is too small for mid-to-long-term frozen storage; therefore, a working solution was prepared from a 7 μL aliquot diluted with 93 μL molecular-grade water, from which 14.3 μL (20 ng) was used for each library.

Twist cfDNA Library Preparation Kit, using 9 PCR cycles. Library length distribution and amount were measured using Agilent TapeStation D1000 Screen Tape (Agilent, USA). 1,600 ng of each library was pooled into an 8-plex pool, yielding 12,800 ng. Capture was performed using the pancreatic cancer and ovarian cancer sub-panels. All 12,800 ng of 8-plex pool were used for capture with the panel and the Twist Target Enrichment Standard Hybridization v2 capture reagents. 16 PCR cycles were used to amplify the library pool after capture.

Previous experience with UMI-based error correction using kits from other vendors suggested that raw sequencing output would need to be at least 10 times the final number of sequences remaining after error correction. The initial exploration sequencing run on the Illumina NextSeq 1000 P2 flow cell with 2 × 100 bp paired-end reads was specified to have a sequencing output of 3.33 Gbp per library. This meant a raw coverage in the pancreas panel libraries of 66,600x. After bioinformatic error-correction, the number of reads per family was found to be rather high, and the previously specified sequencing output was found to be too low to distinguish true mutations from unspecific and probably false-positive variant-calls in the 0.1% Twist cfDNA reference sample replicates.

### Step 2: Second experiment at user site

In a second experiment, the amount of cfDNA was increased to 40 ng. The number of PCR cycles was reduced to 8 cycles in the library preparation. 2,000 ng of each library was pooled into an 8-plex pool for subsequent capture. The number of PCR cycles in the capture reaction was reduced to 14 cycles. The sequencing run on the Illumina NovaSeq 6000 S4 flowcell with 2 × 150 bp paired-end reads was specified to yield a sequencing output of 10 Gbp per library.

### Step 3: Third experiment: sequencing at subcontractor site MGI

In a third experiment, the library pools from the first and second runs were sequenced on the MGI DNBSEQ-G400 instrument (MGI, China) and specified to yield a sequencing output of 5 Gbp per pancreas panel library. For MGI sequencing, 50 ng of each pooled Twist DNA library was converted into an MGI-compatible single-stranded circular (ssCir) library using the MGIEasy Universal Library Conversion Kit (App-A, MGI Tech Co., Ltd.), following the manufacturer’s instructions. Briefly, the linear double-stranded Twist libraries were subjected to Adapter Conversion PCR (5 cycles) to add MGI-compatible adapter sequences, followed by magnetic bead purification to remove residual primers and short fragments.

The purified products were then heat-denatured to obtain single-stranded DNA, which was circularized to form ssCir libraries. Non-circular molecules were enzymatically digested, and the resulting ssCir libraries were purified again. Subsequently, DNA nanoballs (DNBs) were generated by rolling-circle amplification and loaded onto DNBSEQ-G400 flow cells for sequencing according to standard MGI protocols, using paired-end 150 bp (PE150) read mode.

## 3. Results

### Sequencing metrics

**Figure 1.**
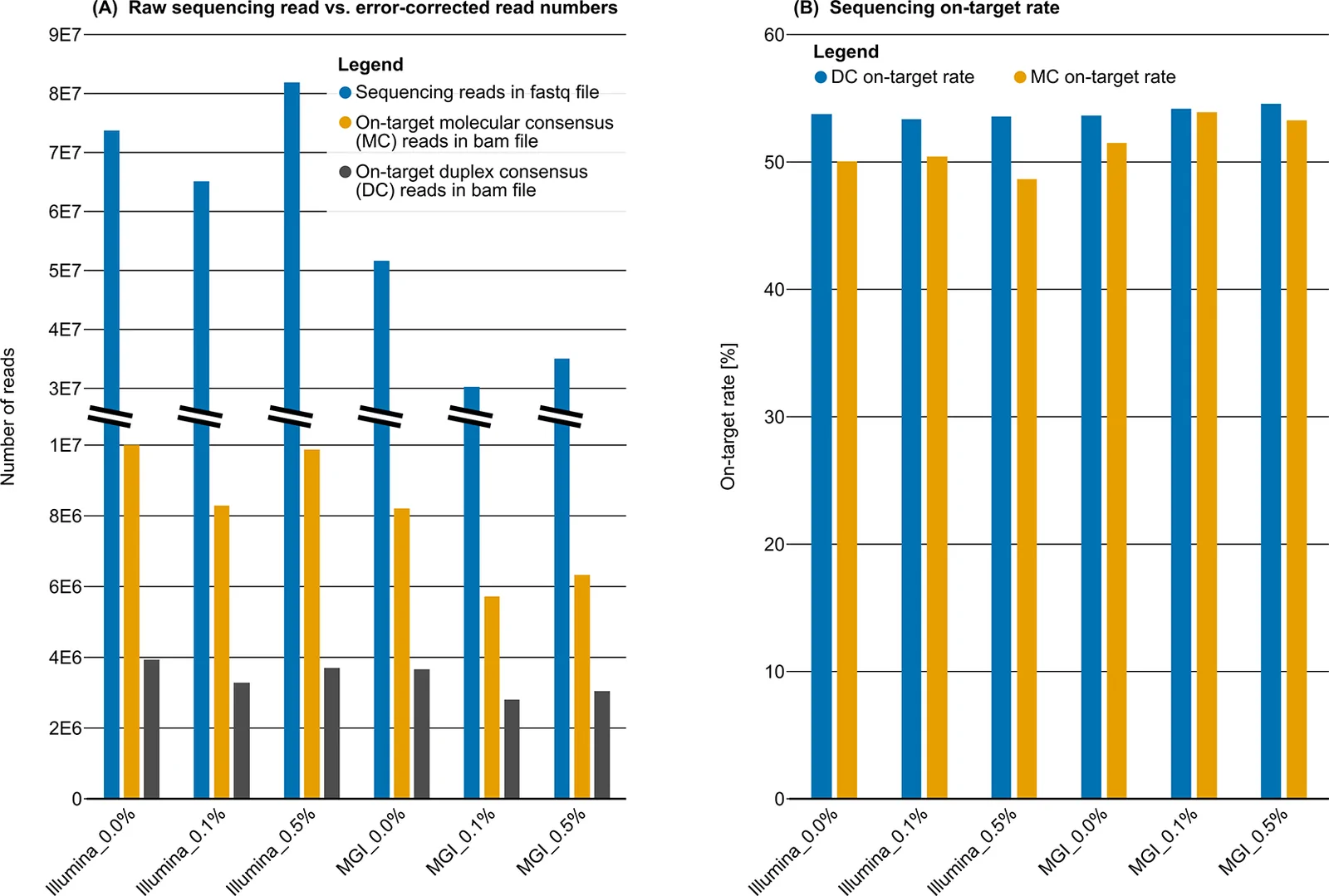
Sequencing depth and on-target performance of the pancreas panel. This figure summarizes the sequencing depth required to achieve adequate molecular consensus (MC) and duplex consensus (DC) read recovery for a sequencing on-target rate of >50%. (A) Total sequencing output for Illumina and MGI platforms across three cfDNA Pan-Cancer Reference Standards with variant allele frequencies (VAFs) of 0.0% (negative control), 0.1%, and 0.5%. Note the break in the x-axis scale. For each sequencing technology and reference sample, the total number of reads generated (raw FASTQ) and the number of aligned on-target reads retained in MC and DC BAM files are shown. (B) On-target rates following hybridization-capture enrichment, illustrated by sequencing technology, reference sample, and consensus level (MC vs. DC).

Sequencing of enriched libraries yielded a mean of 56.3 million raw paired-end reads per sample (range: 30.2–81.9 million), corresponding to an average output of 8.4 Gb per library. As shown in Figure 1, the sequencing depth of the original Illumina libraries was on average 88.8% higher than that obtained after conversion and sequencing on the MGI platform, which is approximately in line with expectations from the 10 Gbp sequencing output specified in the Illumina experiment versus the 5 Gbp sequencing output in the MGI experiment.

After alignment and hybridization capture, a mean on-target rate of 52.4% (range: 48.7–54.2%) was achieved. This resulted in an average molecular consensus (MC) coverage of 13,540x (range: 10,213–16,476x) and a duplex consensus (DC) coverage of 6,036x (range: 5,011–6,975x) across the targeted regions. These coverage levels were sufficient to detect variants at low VAFs.

### Accuracy and sensitivity of the pancreatic cancer panel

**Figure 2.**
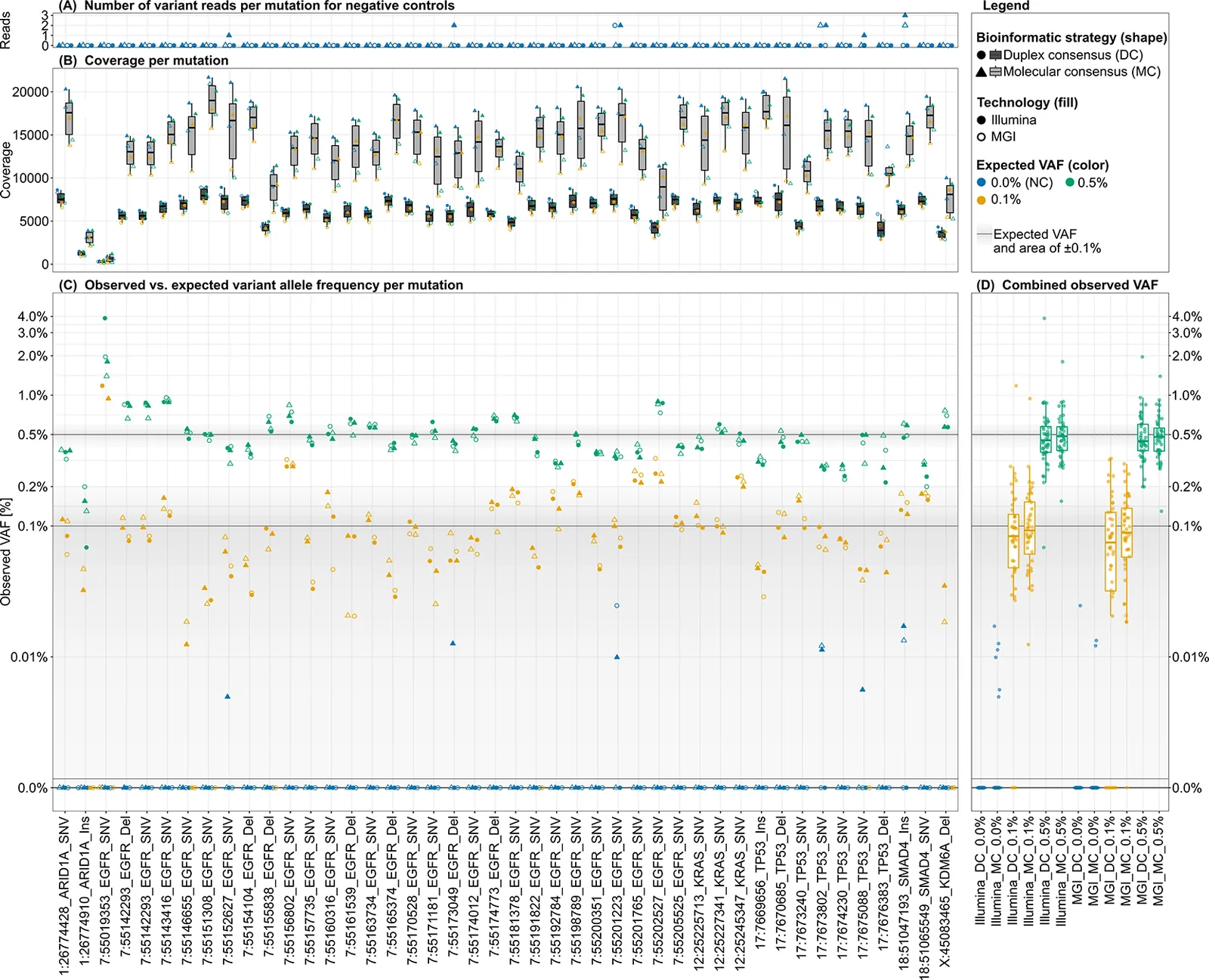
Technical assessment of sensitivity and specificity for mutation detection using a pancreatic cancer panel. The figure summarizes variant detection performance across all 44 mutation positions included in the pancreatic cancer panel and reference samples. Shown are the number of variant reads, sequencing coverage, and observed versus expected variant allele frequency (VAF). Data are stratified by bioinformatic strategy (duplex and molecular consensus; circles and triangles), sequencing technology (Illumina and MGI; filled and open symbols), and cfDNA Pan-Cancer Reference Standards allele frequency (0.0%, 0.1%, and 0.5%; blue, orange, and green, respectively). A combined analysis across all panel positions is also shown. (A) Number of variant reads detected at each mutation position in the 0.0% reference (negative control). Any detected signal in this panel represents a false-positive event. (B) Sequencing coverage at each mutation position across all reference samples and experimental conditions. (C) Observed VAF for each experiment. Expected VAFs for each reference sample are indicated by solid grey lines, with the ±0.1% VAF range shown as a shaded grey area.

Assay performance for the pancreatic cancer panel was evaluated using the cfDNA Pan-Cancer Reference Standards, which comprise 44 documented mutations within the panel’s target regions. Key performance characteristics are summarized in Figure 2.

False positive signals observed in the 0.0% negative control reference sample are shown in Figure 2A. Eight false positive events were detected in total, seven of which occurred in MC and one in a DC experiment sequenced with MGI. The number of supporting variant reads for these false positives was three reads for MC and two reads for DC.

Coverage at variant loci is presented in Figure 2B. Median coverage was approximately 12,500× for MC and 5,000× for DC analyses, with relatively uniform distribution across most loci. Two loci, chr1:26774910_ARID1A_Ins and chr7:55019353_EGFR_SNV, showed consistently reduced coverage and were reported to the panel manufacturer to address them in subsequent panel versions. The observed MC-to-DC coverage ratio of approximately 2.5 exceeds the theoretically optimal expectation of 2.0, indicating the removal of 25% additional reads during DC construction due to missing the read representing the respective complementary strand.

Observed VAFs for all reference mutations are shown in Figure 2C. All expected 0.5% VAF variants were detected. Of the 176 expected 0.1% VAF signals, 12 (6.8%) were not detected. Excluding the two previously described lowly covered loci, the number of missed 0.1% signals is reduced to eight. Two additional missed variants were located in the X-chromosomal gene *KDM6A*, which exhibited only approximately half the coverage of autosomal loci, consistent with the male reference standard genotype. Of the 12 missed signals, nine occurred in MGI experiments and three in Illumina experiments; when excluding previously described low-coverage and X-chromosomal loci, five signals were missed in the MGI experiments and none in Illumina experiments.

Figure 2D summarizes VAF distributions across all variants, stratified by sequencing technology and bioinformatic strategy. For 0.1% VAF reference samples, interquartile ranges (IQRs) were fully contained within the ±0.1% VAF window around the expected value. For the 0.5% reference samples, the upper quartiles remained within this range, whereas the lower quartiles extended slightly below −0.1%. Overall, MC-derived VAFs were more closely centered around expected values than DC-derived VAFs, while no strong systematic differences were observed between sequencing platforms. A modest trend toward improved performance with Illumina sequencing should be interpreted in the context of almost double the sequencing depth (88.8% higher) in the Illumina experiment and additional PCR cycles introduced during the Illumina-to-MGI library conversion.

Applying a minimum coverage threshold of 4,000x reduced the number of missed 0.1% variants from 12 to five (one Illumina DC and four MGI DC). Across all experiments, nine false positive events were observed, predominantly in MC analyses (six Illumina and two MGI), whereas only a single DC false positive was detected (MGI, two supporting reads).

### Accuracy and sensitivity of the ovarian cancer panel

**Figure 3.**
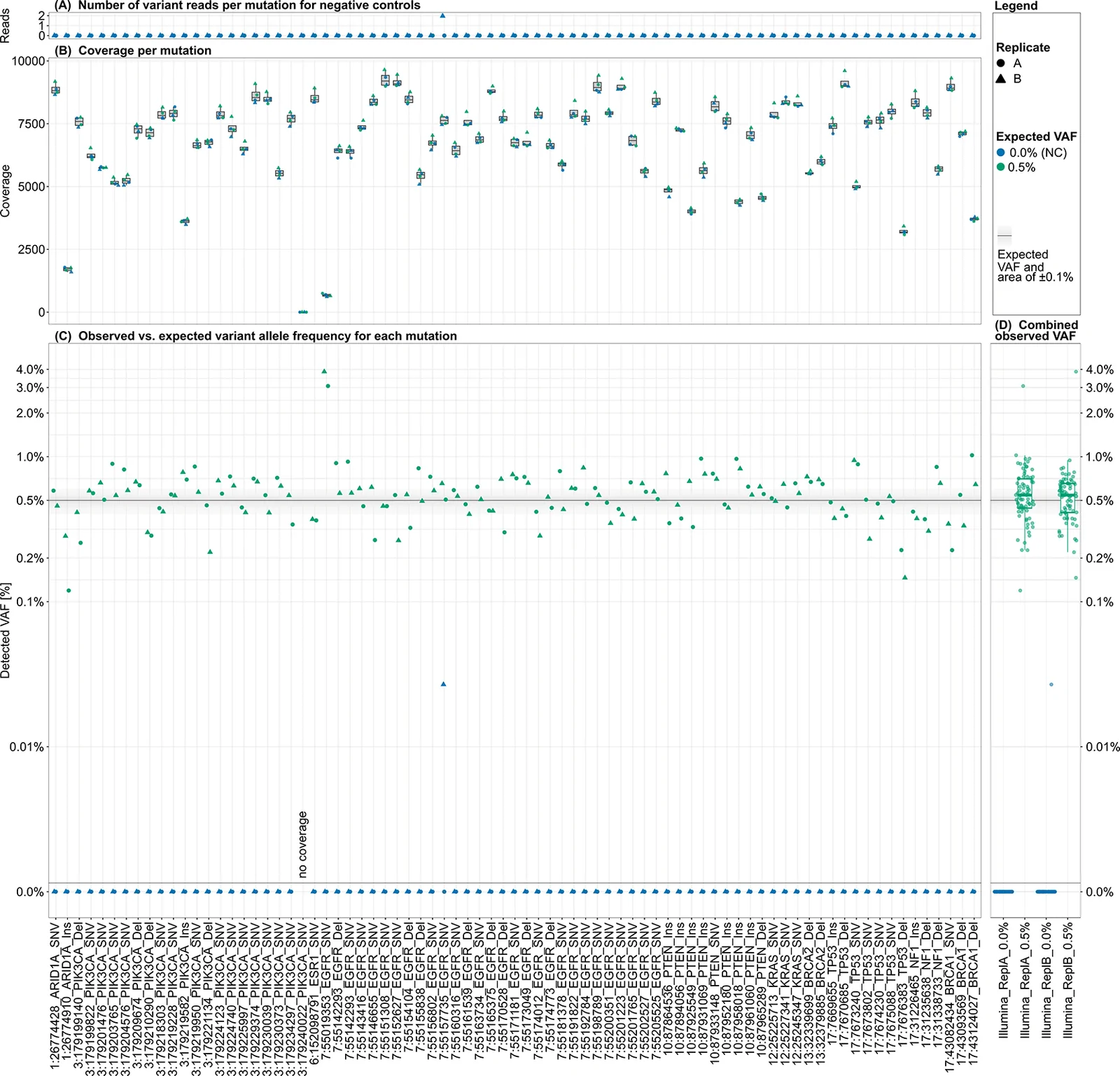
Technical assessment of sensitivity and specificity for mutation detection using an ovarian cancer panel. The figure summarizes variant detection performance across all 79 mutation positions included in the ovarian cancer panel and reference samples. Shown are the number of variant reads, sequencing coverage, and observed versus expected variant allele frequency (VAF). Data are stratified by technical replicates (circles and triangles) and cfDNA Pan-Cancer Reference Standards allele frequency (0.0% and 0.5%; blue and green, respectively). A combined analysis across all panel positions is also shown. (A) Number of variant reads detected at each mutation position in the 0.0% reference (negative control). Any detected signal in this panel represents a false-positive event. (B) Sequencing coverage at each mutation position across the two reference samples and technical replicates. (C) Observed VAF for each experiment. The expected VAF for the 0.5% reference sample is indicated by a solid grey line, with the ±0.1% VAF range shown as a shaded grey area.

The performance of the ovarian cancer panel was evaluated using 79 documented reference mutations located within the panel’s target regions ( Figure 3). Based on results from the pancreatic panel analysis, Illumina sequencing was selected, and the DC bioinformatic strategy was applied to maximize specificity. Because DC coverage was below 4,000×, the threshold defined in the previous subchapter, sequencing depth was considered insufficient for reliable assessment of variants at 0.1% VAF in 7 of the 79 reference loci (8.9%). Consequently, downstream analyses were restricted to the 0.5% reference samples analyzed in technical replicates.

False positive signals in the 0.0% negative control sample are shown in Figure 3A. Only one false positive event (0.63%) was observed, supported by two variant reads.

Coverage across variant loci is shown in Figure 3B. Coverage was generally uniform, with a mean of 7,277× (range: 0–9,647×). Three loci exhibited substantially reduced coverage: chr1:26774910_ARID1A_Ins and chr7:55019353_EGFR_SNV, consistent with the pancreatic panel results, and an additional locus, chr3:179240022_PIK3CA_SNV, which lacked coverage with sequencing reads entirely. As communicated by the manufacturer, this locus is addressed in the updated panel design version.

Observed VAFs for the 0.5% reference variants are shown in Figure 3C. All expected variants were detected, except at the locus with no sequencing coverage. Combined VAF distributions for all loci and replicates are presented in Figure 3D. The lower quartiles of the observed VAFs fell within −0.1% of the expected value, whereas the upper quartiles extended beyond +0.1%. Notably, the observed VAF distribution of replicate B was distinctly different, with the distribution shifted to lower VAFs. This suggests that differences between replicates may be larger than the differences observed between sequencing technologies.

### Bioinformatic workflow

We here publish the complete bioinformatic strategy used to generate error-corrected UMI-based MC and DC data. All software tools, including version information, and all parameters used during data processing are reported. A comprehensive resource describing the full workflow, including software, parameters, and executed commands, is provided in the **Supplementary File S1**.

## 4. Discussion

In this study, we evaluated the technical feasibility of detecting low-level variants using two targeted enrichment panels for pancreatic and ovarian cancer, with VAFs ranging from 0.5% down to 0.1%. By systematically comparing two sequencing platforms and two bioinformatic strategies, we provide practical recommendations regarding filtering criteria, sequencing depth, and analytical workflows for reliable low-level VAF variant detection.

Across 123 reference variants covered by the two panels, the overall specificity of the assay developed by the Instand-NGS4P subcontractor consortium TPM was high. Only two false-positive signals were observed with the DC bioinformatic strategy, which could be effectively mitigated by applying a minimum variant-read-count threshold. Notably, most false positives originated from the MC bioinformatic strategy, which was included as a more sensitive alternative to the DC approach. Specifically, eight of nine false positives detected in the pancreatic panel occurred in MC analyses, whereas only two false positives were identified using the DC strategy across all 123 loci. These findings highlight a trade-off between sensitivity and specificity, with MC offering increased sensitivity at the high cost of reduced specificity.

Sensitivity analyses revealed platform-dependent differences. While DC sensitivity was comparable across strategies for Illumina sequencing, it was reduced in MGI experiments, where eight 0.1% VAF mutations were missed compared with three variants on Illumina. In contrast, the MC strategy detected all reference variants in Illumina datasets and missed only a single 0.1% variant in MGI data. When excluding loci with insufficient sequencing depth, primarily due to uneven panel coverage or X-chromosomal localization in the genetically male reference sample, no variants were missed using Illumina DC, whereas five variants remained undetected in MGI datasets.

The observed performance differences between Illumina and MGI platforms are at least partially attributable to the substantially higher sequencing depth specified for the Illumina experiment (88.8% higher) and to the additional five PCR cycles required for Illumina-to-MGI library conversion, which may introduce or amplify polymerase-induced artifacts. The Illumina sequencing results for adequately covered loci indicate that the bioinformatic DC strategy provides superior specificity without compromising sensitivity relative to the bioinformatic MC strategy.

Coverage uniformity of the panel designs was generally satisfactory, although three loci consistently showed reduced depth. According to the manufacturer, these design-related limitations will be addressed in future panel iterations. Based on empirical observations, we propose a minimum depth of 4,000x duplex-consensus reads to ensure robust detection of variants with a 0.1% VAF using Illumina sequencing. Therefore, the approach we here present is an optimal compromise between affordability and sufficient sensitivity and specificity.

Under the selected analytical conditions (DC using Illumina sequencing with a minimum depth of 4,000× DC reads), the assay demonstrated high analytical performance. When sensitivity and specificity were evaluated without applying an additional filter requiring at least 3 supporting variant reads, the assay achieved a specificity of 98.37%, calculated from all observed false-positive calls across the pancreatic and ovarian datasets. Sensitivity was 100%, as all variants present at a variant allele frequency of 0.1% in the pancreatic panel were consistently detected.

Compared with other recently published NGS-based methodologies, the approach developed by the TPM consortium has a limit of detection (LoD) of ≤0.1%. Although alternative strategies have reported LoDs as low as 0.001% ^12^ or even 0.0001%, ^13^ these gains in analytical sensitivity are achieved at the cost of substantially increased complexity and expense. Specifically, such approaches rely on bespoke hybridization panel design for each individual sample or require very deep sequencing, comparable to whole-genome sequencing at approximately 60x coverage, representing a 19.2-fold increase in sequencing depth relative to the method presented here, thereby limiting their economic feasibility for routine application.

Overall, this study establishes a closed, transferable, and reproducible workflow for low-level VAF variant detection using standardized reference materials. Developed within the Instand-NGS4P project, this approach represents a promising technical foundation for future MRD applications and clinical translation. By directly comparing sequencing platforms and bioinformatic strategies, our work provides a transparent assessment of methodological trade-offs relevant to low-level variant detection. The technical performance observed here is consistent with the favorable evaluation at the conclusion of Instand-NGS4P Phase III, further supporting the robustness of the TWIST/Platomics/MGI consortium’s approach. Moreover, by incorporating feedback from the European Commission’s final review, this study emphasizes both the clinical relevance and the degree of standardization achieved for the MRD panels developed within this EU-funded framework.

Several limitations should be acknowledged. This study was intentionally restricted to technical performance metrics and standardized reference samples. In addition, the workflow was assessed using a cfDNA input of 40 ng, which may not always be obtainable from patient plasma samples in routine clinical practice. Panel designs may evolve to improve coverage uniformity or to incorporate additional genes as therapeutic landscapes change. Importantly, prior to clinical implementation, the described workflows and panels must be validated in real-world clinical studies using patient material and undergo lab-developed test (LDT) accreditation or IVDR certification based on those data.

Beyond the results presented here for one representative solution, the broader Instand-NGS4P project subjected each final-phase approach to multi-site evaluation and evaluation from technical, clinical, and patient perspectives, with further manuscripts to follow. Taken together, these efforts culminated in a project that, according to the European Commission’s General Project Review, fully achieved its objectives and delivered exceptional results with significant immediate or potential impact.

## Ethics and consent

No human samples were used in this study. All experiments were performed using synthetic reference materials (synthetic Twist cfDNA Pan-Cancer Reference Standard v2). Because no human participants or identifiable human biological materials were involved, ethics approval and informed consent were not required.

## Software availability

A detailed description of all software and software versions, together with a generalized script containing all commands and parameters used for the bioinformatic analyses in this manuscript, is provided in **Supplementary File S1**.

## Data Availability

The aligned bam files generated and analyzed during the current study are publicly available in the European Nucleotide Archive (ENA) under accession number PRJEB108992. ^14^ The supplementary materials, including a detailed description of the bioinformatic workflow (steiert_et_al_supplementary_file_S1.pdf ) and the list of genes covered by the ovarian and pancreas panels (steiert_et_al_supplementary_tables.xlsx), are available at https://doi.org/10.5281/zenodo.18967866. ^15^ Data are available under the terms of the Creative Commons Attribution 4.0 International (CC-BY 4.0).

## References

[1] Dongen JJ van Velden VH van der Brüggemann M : Minimal residual disease diagnostics in acute lymphoblastic leukemia: need for sensitive, fast, and standardized technologies. *Blood.* 2015;125(26):3996–4009. 10.1182/blood-2015-03-580027 25999452 PMC4490298

[2] Tie J Wang Y Tomasetti C : Circulating tumor DNA analysis detects minimal residual disease and predicts recurrence in patients with stage II colon cancer. *Sci. Transl. Med.* 2016;8. 10.1126/scitranslmed.aaf6219 PMC5346159 27384348

[3] Gale D Heider K Ruiz-Valdepenas A : Residual ctDNA after treatment predicts early relapse in patients with early-stage non-small cell lung cancer. *Ann. Oncol.* 2022;33(5):500–510. 10.1016/j.annonc.2022.02.007 35306155 PMC9067454

[4] Garcia-Murillas I Schiavon G Weigelt B : Mutation tracking in circulating tumor DNA predicts relapse in early breast cancer. *Sci. Transl. Med.* 2015;7. 10.1126/scitranslmed.aab0021 26311728

[5] Tan L Sandhu S Lee RJ : Prediction and monitoring of relapse in stage III melanoma using circulating tumor DNA. *Ann. Oncol.* 2019;30(5):804–814. 10.1093/annonc/mdz048 30838379 PMC6551451

[6] Christensen E Birkenkamp-Demtröder K Sethi H : Early Detection of Metastatic Relapse and Monitoring of Therapeutic Efficacy by Ultra-Deep Sequencing of Plasma Cell-Free DNA in Patients With Urothelial Bladder Carcinoma. *J. Clin. Oncol.* 2019;37(18):1547–1557. 10.1200/JCO.18.02052 31059311

[7] Fei X Du X Gong Y : Early Plasma Circulating Tumor DNA as a Potential Biomarker of Disease Recurrence in Non-metastatic Prostate Cancer. *Cancer Res. Treat.* 2023;55(3):969–977. 10.4143/crt.2022.1557 36915250 PMC10372593

[8] Jiang J Ye S Xu Y : Circulating Tumor DNA as a Potential Marker to Detect Minimal Residual Disease and Predict Recurrence in Pancreatic Cancer. *Front. Oncol.* 2020;10:1220. 10.3389/fonc.2020.01220 32850360 PMC7406781

[9] OECD, European Commission: *EU Country Cancer Profiles Synthesis Report 2025.* Paris: OECD Publishing;2025. 10.1787/20ef03e1-en

[10] Pengelly RJ Gibson J Andreoletti G : A SNP profiling panel for sample tracking in whole-exome sequencing studies. *Genome Med.* 2013;5(9):89. 10.1186/gm492 24070238 PMC3978886

[11] Pengelly RJ Gibson J Andreoletti G : Erratum to: A SNP profiling panel for sample tracking in whole-exome sequencing studies. *Genome Med.* 2015;7(5):44. 25949530 10.1186/s13073-015-0163-1 PMC4422541

[12] Heo S Ham SK Lee H : Analytical validation of a hybrid-approach combining tumor-informed and tumor-agnostic bespoke ctDNA panel assay for the sensitive detection of minimal residual disease. *PLoS One.* 2025;20(11):e0334282. 10.1371/journal.pone.0334282 41212866 PMC12599964

[13] Li X Liu T Bacchiocchi A : Ultra-sensitive molecular residual disease detection through whole genome sequencing with single-read error correction. *EMBO Mol. Med.* 2024;16(9):2188–2209. 10.1038/s44321-024-00115-0 39164471 PMC11393307

[14] Steiert TA Altrock PM Künstner A : Aligned bam files: Measurable Residual Disease method development for pancreatic and ovarian cancer detection. *Eur. Nucleot. Arch.* 2026. Reference Source

[15] Steiert TA Altrock PM Künstner A : Supplementary materials: Method development for pancreatic and ovarian cancer baseline ctDNA detection and measurable residual disease monitoring. *Zenodo.* 2026. 10.5281/zenodo.18967866

