# Supplementary material for: Method development for pancreatic and ovarian cancer baseline ctDNA detection and measurable residual disease monitoring
Source: Open Res Eur. 2026 Jul 13;6:87. Originally published 2026 Apr 3. [Version 2] doi: 10.12688/openreseurope.23403.2 (PMC13560845; doi:10.12688/openreseurope.23403.2)
Supplement: Peer review history

# Reviewer report: Reviewer response for version 2

**DOI:** 10.21956/openreseurope.26519.r77329

**Published:** 2026-08-03

**Authors:** Yibo Dai

**Affiliations:** 1 The University of Texas MD Anderson Cancer Center, Houston, USA

The authors' careful revision with additional descriptions and justifications is appreciated. I don't have further concerns regarding this manuscript.

Is the rationale for developing the new method (or application) clearly explained?

Yes

Is the description of the method technically sound?

Yes

Are the conclusions about the method and its performance adequately supported by the findings presented in the article?

Yes

If any results are presented, are all the source data underlying the results available to ensure full reproducibility?

No

Are sufficient details provided to allow replication of the method development and its use by others?

Partly

Reviewer Expertise:

Bioinformatics, oncology

I confirm that I have read this submission and believe that I have an appropriate level of expertise to confirm that it is of an acceptable scientific standard.

# Reviewer report: Reviewer response for version 2

**DOI:** 10.21956/openreseurope.26519.r77330

**Published:** 2026-07-17

**Authors:** Myron G. Best

**Affiliations:** 1 Amsterdam UMC Location Vrije Universiteit Amsterdam, Amsterdam, The Netherlands

The authors have (experimentally) answered all my comments.

Is the rationale for developing the new method (or application) clearly explained?

Yes

Is the description of the method technically sound?

Yes

Are the conclusions about the method and its performance adequately supported by the findings presented in the article?

Yes

If any results are presented, are all the source data underlying the results available to ensure full reproducibility?

Yes

Are sufficient details provided to allow replication of the method development and its use by others?

Yes

Reviewer Expertise:

Liquid biopsies, translational research, sequencing

I confirm that I have read this submission and believe that I have an appropriate level of expertise to confirm that it is of an acceptable scientific standard.

# Reviewer report: Reviewer response for version 1

**DOI:** 10.21956/openreseurope.25321.r72154

**Published:** 2026-05-07

**Authors:** Yibo Dai

**Affiliations:** 1 The University of Texas MD Anderson Cancer Center, Houston, USA

This study presented a technical analysis of MRD detection by a targeted panel for pancreatic and ovarian cancers. The inclusion of detailed bioinformatic evaluation data is appreciated. Nevertheless, there are a few key points that need to be addressed to enhance the manuscript and its clinical relevance, as detailed below.

1. It is not clear why the authors put pancreatic and ovarian cancers together in the same study, since the two disease are very different is cells of origin, pathogenesis, pathology and clinical course. The study design needs to be justified.
2. In the manuscript, for the panel design, the author stated that “The consortium experts specified gene lists for MRD detection in pancreatic cancer ( Table S1) and ovarian cancer ( Table S2), based on publicly available data from The Cancer Genome Atlas on the most recurrently mutated gene in these cancer entities.” This sounds a bit too general. Would it be possible to provide more rigorous data to support the current panel design?
3. It would be helpful to demonstrate the clinical value of MRD detection by the current panel and analysis workflow, such as correlation with disease stages, survival, therapy response monitoring, etc. This is critical for its potential integration into clinical practice.

Is the rationale for developing the new method (or application) clearly explained?

Yes

Is the description of the method technically sound?

Yes

Are the conclusions about the method and its performance adequately supported by the findings presented in the article?

Yes

If any results are presented, are all the source data underlying the results available to ensure full reproducibility?

No

Are sufficient details provided to allow replication of the method development and its use by others?

Partly

Reviewer Expertise:

Bioinformatics, oncology

I confirm that I have read this submission and believe that I have an appropriate level of expertise to confirm that it is of an acceptable scientific standard, however I have significant reservations, as outlined above.

# Response

**Published:** 2026-07-02

**Authors:** Tim Alexander Steiert

**Reviewer #2, Yibo Dai**, The University of Texas MD Anderson Cancer Center, Houston, USA: This study presented a technical analysis of MRD detection by a targeted panel for pancreatic and ovarian cancers. The inclusion of detailed bioinformatic evaluation data is appreciated. Nevertheless, there are a few key points that need to be addressed to enhance the manuscript and its clinical relevance, as detailed below. We thank the reviewer for the thorough evaluation of our manuscript and appreciate the constructive and critical feedback provided. [#2-01] It is not clear why the authors put pancreatic and ovarian cancers together in the same study, since the two disease are very different is cells of origin, pathogenesis, pathology and clinical course. The study design needs to be justified.

 Response:  Thank you for this valuable comment. We agree that the technical focus of the manuscript and its relationship to the broader EU project were not sufficiently explained. Although the Instand-NGS4P project addressed a broad range of cancer entities, the present manuscript focuses on the technical evaluation of two MRD panel solutions for pancreatic and ovarian cancer, which are of particular interest at the authors’ institutions. To provide this context, we have expanded the Introduction with a brief description of the Instand-NGS4P project. The original manuscript only mentioned the project call and grant information, which did not adequately convey the project's objectives or the rationale for the present work. The added text clarifies that this manuscript reports the technical evaluation of prototype developments generated within the framework of a European pre-commercial procurement (PCP) project. The following paragraph has been added to the Introduction: "Instand‑NGS4P (https://cordis.europa.eu/project/id/874719, https://www.instandngs4p.eu) was a PCP project running from 2020-2025 that included many European medical centers, patient advocacy groups (ECPC, FAVO), a standards organization (DIN), and partners linked to major European research infrastructures (e.g., BBMRI‑ERIC, ELIXIR). Its aims were to develop a European standard and the respective modular technical-clinical framework for NGS workflows for routine diagnostics and to engage with subcontractors, who were selected in an open tendering process and contracted to develop pre-commercial innovative modular prototypes or developments (e.g. reference materials, library preparation kits, or analysis software) in the domain of NGS examinations for common and rare cancers, in children and adults. The consortium selected 8 contractors in the last competitive round of tendering, from the original 24 tenders received. We here report on our technical testing of two of the MRD custom panel solutions and the cfDNA reference standards that were developed during the project run time"

 [#2-02] In the manuscript, for the panel design, the author stated that “The consortium experts specified gene lists for MRD detection in pancreatic cancer ( Table S1) and ovarian cancer ( Table S2), based on publicly available data from The Cancer Genome Atlas on the most recurrently mutated gene in these cancer entities.” This sounds a bit too general. Would it be possible to provide more rigorous data to support the current panel design?

 Response: Thank you for this helpful comment. We agree that this aspect required further and more rigorous clarification. To address the reviewer's comment, we have added the following sentences: "We used the freely available NIH National Cancer Institute's GDC Data Portal to retrieve the TCGA Ovarian Serous Cystadenocarcinoma (TCGA-OV), or the TCGA Pancreatic Adenocarcinoma (TCGA-PAAD) mutation data, respectively. We used the GDC portal's Analysis Center with the OncoMatrix App to display the most recurrently mutated genes. For each of the two panels, we selected the most frequently mutated gene, e.g. *TP53* for TCGA-OV, together with additional genes that were recurrently mutated in the TCGA cohort among cases lacking *TP53* mutations. For the pancreatic cancer panel we included *TP53* and *DNMT3A* as CHIP-and-also-cancer genes, based on two of our previous studies with colon inflammation/cancer and esophageal cancer that may develop in inflamed tissue (PMIDs: 36271073, 37445849)."   [#2-03] It would be helpful to demonstrate the clinical value of MRD detection by the current panel and analysis workflow, such as correlation with disease stages, survival, therapy response monitoring, etc. This is critical for its potential integration into clinical practice. Thank you for this comment, which supports our recently submitted proposal application for the follow-on EU project Instand-NGS4P-II for integration into clinical practice and demonstration of the clinical value. We have submitted the follow-on project proposal as a PPI-project (public procurement of innovative solutions) in response to the corresponding call for proposals by the European Commission.

# Reviewer report: Reviewer response for version 1

**DOI:** 10.21956/openreseurope.25321.r72658

**Published:** 2026-04-30

**Authors:** Myron G. Best

**Affiliations:** 1 Amsterdam UMC Location Vrije Universiteit Amsterdam, Amsterdam, The Netherlands

In this work, the authors construct a reference mutation panel from publicly available data, and develop an MRD assay for pancreatic and ovarian cancer ctDNA analysis. This is done as part of an effort to stimulate EU-based biotechnology. They take advantage of a reference DNA sample which they subject to MGI and Illumina sequencing protocols, and assess expected versus observed VAF-accuracies. In all, the work is sound and well described. The conclusions are logical and the discussion balanced.

 My comments:

- My primary comment is the input amount of DNA. As discussed in their discussion section, 40 ng cannot always be reached in cancer patients, let stand alone those in minimal tumour volume MRD setting. This can have a significant impact on the assay’s sensitivity and may overestimate current performance. To make the results more relevant, the assay’s performance should also be tested at lower cfDNA input amounts.
- They authors have put a significant effort into selection of most relevant mutations in the two tumour types. It remains unclear to me how many of the potential mutations they started with ended up in their final panel’s selection.
- Were analysis in Fig. 1 performed in duplo/triplo?
- Fig. 3D and its text: ‘The lower quartiles of the observed VAFs fell within 0.1% of the expected value, whereas the upper quartiles extended beyond +0.1%. Notably, the observed VAF distribution of replicate B was distinctly different, with the distribution shifted to lower VAFs’. I do not deduce this from the Fig 3D boxplots, they seem to overlap. Is this phrasing correct?

Is the rationale for developing the new method (or application) clearly explained?

Yes

Is the description of the method technically sound?

Yes

Are the conclusions about the method and its performance adequately supported by the findings presented in the article?

Yes

If any results are presented, are all the source data underlying the results available to ensure full reproducibility?

Yes

Are sufficient details provided to allow replication of the method development and its use by others?

Yes

Reviewer Expertise:

Liquid biopsies, translational research, sequencing

I confirm that I have read this submission and believe that I have an appropriate level of expertise to confirm that it is of an acceptable scientific standard, however I have significant reservations, as outlined above.

# Response

**Published:** 2026-07-02

**Authors:** Tim Alexander Steiert

**Reviewer #1, Myron G. Best**, Amsterdam UMC Location Vrije Universiteit Amsterdam, Amsterdam, The Netherlands: In this work, the authors construct a reference mutation panel from publicly available data, and develop an MRD assay for pancreatic and ovarian cancer ctDNA analysis. This is done as part of an effort to stimulate EU-based biotechnology. They take advantage of a reference DNA sample which they subject to MGI and Illumina sequencing protocols, and assess expected versus observed VAF-accuracies. In all, the work is sound and well described. The conclusions are logical and the discussion balanced. We thank the reviewer for the generally positive and balanced assessment of our work. My comments:

 [#1-01] My primary comment is the input amount of DNA. As discussed in their discussion section, 40 ng cannot always be reached in cancer patients, let stand alone those in minimal tumour volume MRD setting. This can have a significant impact on the assay’s sensitivity and may overestimate current performance. To make the results more relevant, the assay’s performance should also be tested at lower cfDNA input amounts.

 Response: Thank you for raising this important point, which is highly relevant to clinical practice, where our clinical laboratories are currently requesting two or even three blood tubes for a cfDNA-based examination. We agree that samples containing less than 40 ng of cfDNA are encountered in a subset of patients. To address the reviewer's concern, we performed additional experiments using the same reference DNA sample at a reduced input amount. As reported in the literature [PMID: 36915242], a standard 10 mL blood draw from a cancer patient typically yields 4-5 mL of plasma containing approximately 5-10 ng cfDNA per mL of plasma, corresponding to a total yield of 20-50 ng. We therefore selected 20 ng, representing the lower end of this range, for additional evaluation experiments. Assay sensitivity was assessed under these conditions, and the corresponding results and their implications have been incorporated into the Materials and Methods, the Results and the Discussion sections of the revised manuscript.

 [#1-02] They authors have put a significant effort into selection of most relevant mutations in the two tumour types. It remains unclear to me how many of the potential mutations they started with ended up in their final panel’s selection.

 Thank you for this comment, which partly is reflected by the second comment of the second reviewer

 [#2-02]. To address the comment regarding the original number of mutations documented for the TWIST cfDNA Pan-Cancer Reference Standards, we added the following sentence into the Materials and Methods section: "The 0.1% and 0.5% VAF reference standards contain the following documented synthetic variants at the respective expected allele frequencies: 225 SNVs, 49 insertions, 167 deletions, and 15 SVs." The reference mutations analyzed in this study comprise all variants contained in the cfDNA reference standards that overlap with the genomic target regions of the pancreatic and ovarian panels evaluated in this study. To further clarify the design of the pancreatic and ovarian sub-panels, we have added the following text to the "Instand-NGS4P pancreatic sub-panel and ovarian sub-panel" subsection of the Materials and Methods: "We used the freely available NIH National Cancer Institute's GDC Data Portal to retrieve the TCGA Ovarian Serous Cystadenocarcinoma (TCGA-OV), or the TCGA Pancreatic Adenocarcinoma (TCGA-PAAD) mutation data, respectively. We used the GDC portal's Analysis Center with the OncoMatrix App to display the most recurrently mutated genes. For each of the two panels, we selected the most frequently mutated gene, e.g. *TP53* for TCGA-OV, together with additional genes that were recurrently mutated in the TCGA cohort among cases lacking *TP53* mutations. For the pancreatic cancer panel we included *TP53* and *DNMT3A* as CHIP-and-also-cancer genes, based on two of our previous studies with colon inflammation/cancer and esophageal cancer that may develop in inflamed tissue (PMIDs: 36271073, 37445849).

 [#1-03] Were analysis in Fig. 1 performed in duplo/triplo? Thank you for this helpful comment. To clarify the experimental design, we have added the following statement to the legend of Figure 1: "Library preparations used 40 ng of input reference standard, and each library was aliquoted for sequencing on both the Illumina and MGI platforms." Furthermore, to improve the clarity of the additional experiments performed in response to the reviewer's first comment, we have added the number of technical replicates to panel A of Figure 4

 . [#1-04] Fig. 3D and its text: ‘The lower quartiles of the observed VAFs fell within 0.1% of the expected value, whereas the upper quartiles extended beyond +0.1%. Notably, the observed VAF distribution of replicate B was distinctly different, with the distribution shifted to lower VAFs’. I do not deduce this from the Fig 3D boxplots, they seem to overlap. Is this phrasing correct? Thank you for this comment. We agree that the original wording was too strong given the relatively small differences observed. We have therefore revised the second sentence to read: "Notably, the median VAF in both replicates were very similar, while the quartiles in replicate B were shifted to slightly lower VAFs"
